# Laying Carpets

**Published:** 1854-07

**Authors:** 


### LAYING CARPETS.

Perhaps eight out of every ten of our readers have experienced the annoyance of laying down carpets; have felt the rush of blood to the head—the straining of the nether garments—the unpleasant rapping of the tops of fingers instead of the tops of the tacks, which that employment is heir to. The foreign correspondent of the *Newark Advertiser*, writing from Florence, suggests the basis of a reform which all housekeepers will appreciate and desire. "Here," he says, "iron rings are fastened in the floors when the carpets are laid, and they have hooks in the binding, for which these rings are eyes, so that there is no taking out and nailing in of tacks, and carpets are raised and laid as noiselessly and easy as bed covers." There are a good many people about this time, we imagine, who will approve of the hook and eye system for carpets, and the abolition of the tack-hammers and bruised fingers.—*Troy Whig.*

